# Impact and Diversity of ESBL-Producing *Klebsiella pneumoniae* Recovered from Raw Chicken Meat Samples in Türkiye

**DOI:** 10.3390/antibiotics13010014

**Published:** 2023-12-21

**Authors:** Cemil Kürekci, Özlem Ünaldı, Seyda Şahin, Isidro García-Meniño, Jens Andre Hammerl

**Affiliations:** 1 Department of Food Hygiene and Technology, Faculty of Veterinary Medicine, Hatay Mustafa Kemal University, Hatay 31060, Türkiye; 2 Department of Microbiology Reference Laboratories, General Directorate of Public Health, Ministry of Health, Ankara 06430, Türkiye; ozlem.unaldi@saglik.gov.tr; 3 Department of Food Hygiene and Technology, Faculty of Veterinary Medicine, Sivas Cumhuriyet University, Sivas 58070, Türkiye; seydasahin@cumhuriyet.edu.tr; 4 Laboratorio de Referencia de *Escherichia coli* (LREC), Departamento de Microbioloxía e Parasitoloxía, Facultade de Veterinaria, Universidade de Santiago de Compostela (USC), 27002 Lugo, Spain; isidro.garcia.menino@gmail.com; 5 Department Biological Safety, German Federal Institute for Risk Assessment, 10589 Berlin, Germany; jens-andre.hammerl@bfr.bund.de

**Keywords:** multidrug resistance, *K. pneumoniae*, One Health, food, zoonosis, risk evaluation, consumer protection

## Abstract

The interrelationship between human, animal and environmental sectors leads to the spread of antibiotic resistance due to selective pressures, evolutionary traits and genomic evolution. In particular, the frequent use of antibiotics in livestock inevitably influences the emergence of specific resistance determinants in human strains, associated with reduced treatment options in clinical therapy. In this study, ESBL-producing *Klebsiella pneumoniae* strains isolated from chicken meat samples were evaluated for public health implications in Türkiye. Whole-genome sequencing was used for genetic dissection and phylogenetic comparison of their genomes. The isolates were assigned to four MLST types (ST147, ST37, ST2747 and ST219); two of them were found to represent the ST147 clone associated with severe human infections worldwide. In addition to cephalosporins, high resistance levels to quinolones/fluoroquinolones were identified phenotypically, caused by acquired resistance genes and chromosomal point variations. One isolate was also found to carry the *qacE*∆1 efflux transporter gene, which confers tolerance to quaternary ammonium compounds. The detection of virulence genes (i.e., that coding for enterobactin) associated with the pathogenicity of *K. pneumoniae* suggests a public health impact. Thus, comprehensive information on the occurrence and impact of *K. pneumoniae* from livestock is needed to derive appropriate management strategies for consumer protection. In this study, it was shown that poultry meat serves as a reservoir of clinically emerging multidrug-resistant high-risk clones.

## 1. Introduction

*Klebsiella pneumoniae* is a Gram-negative, rod-shaped, non-motile bacterium representing one of the most common causes of hospital-acquired infections in humans. In the recent past, the rate of *K. pneumoniae* infections increased from 16% (2015, 1617 cases) to 19.9% (2017, 3732 cases) in Türkiye [1,2]. In addition, *K. pneumoniae*-associated infections became a major challenge for human health, due to the development of multidrug resistance (MDR) to antibiotics used for human therapies, i.e., third-generation cephalosporins (cefotaxime, ceftriaxone and ceftazidime) and fluoroquinolones (ciprofloxacin, levofloxacin and ofloxacin) [3]. According to reports obtained through the Central Asian and European Surveillance of Antimicrobial Resistance network, the MDR phenotype was observed in 20% of *K. pneumoniae* in 2015 [1] and increased to 40% in 2018 in Türkiye [3]. 

The production of extended-spectrum β-lactamases (ESBLs) that hydrolyze broad-spectrum cephalosporins and monobactams was identified as the main contributor to this resistance phenotype [4]. These enzymes show remarkable variation in their molecular structure and were assigned to four major classes (A–D) proposed by Ambler [5]. Among the clinically important ESBLs, CTX-M-like enzymes are the most widely distributed enzymes in Enterobacteriaceae, especially in *K. pneumoniae* and *Escherichia coli*, causing healthcare-associated and community-acquired infections in humans worldwide [6]. These enzymes are not solely expressed from the chromosome, but also encoded in mobile genetic elements (especially, plasmids). It has also become apparent that *bla*_CTX-M_-like genes are often accompanied by other antimicrobial/biocide resistance determinants like *aac(6)-Ib-cr*, *bla*_OXA_, *catB*, *tet*, *aadA*, *dfrA17*, *sul* and *qac* genes [7].

Besides their impact on humans, some clonal lineages of *K. pneumoniae* have also been associated with animal infections of the respiratory tract [8,9]. In Egypt, the prevalence of *K. pneumoniae* was reported to be 10% among samples taken from chicken with respiratory diseases [9]. It is also well established that this organism can be present in a variety of foods including raw milk, fresh and processed meat, seafood products and vegetables [10,11,12,13]. Additionally, a recent study carried out by Projahn et al. (2019) demonstrated the contamination and persistence of *K. pneumoniae* in poultry slaughterhouse environments, possibly leading to its spread in the poultry meat chain [14]. Recently, Savin et al. (2021) also highlighted that wastewater from chicken and pig slaughterhouses is a common reservoir for multidrug-resistant *K. pneumoniae* [15]. Despite the isolation of multidrug-resistant *K. pneumoniae*, in particular ESBL-producing strains, from companion and food-producing animals, the role of foods of animal origin in the transmission of *K. pneumoniae* with MDR to humans remains unclear [16]. However, CTX-M-15-producing *K. pneumoniae* from humans was shown to share the same sequence type (ST15) with isolates from companion animals [17,18]. Similarly, Donati et al. (2014) also suggested the likely occurrence of cross-species zoonotic transfer, as *bla*_CTX-M-15_-positive *K. pneumoniae* (ST101) from dogs were found to be closely related to human strains in Italy [19]. 

A few whole-genome sequencing (WGS)-based studies of *K. pneumoniae* strains have set the foundation for understanding the potential contribution of food animals to human infections and the spreading of ESBL resistance [20]. However, the distribution of ESBL-producing *K. pneumoniae* in raw chicken meat samples is unknown in Türkiye. Here, we report on the emergence and genetic basis of ESBL-producing *K. pneumoniae* isolates from chicken meat samples. 

## 2. Results

### 2.1. MDR in K. pneumoniae from Meat 

Out of 200 raw chicken meat samples investigated within a 36-month period in Türkiye between 2017 and 2019, a few ESBL-producing *K. pneumoniae* (n = 5, 2.5%; Supplementary Table S1) strains were selectively recovered and subjected to in-depth phenotypic and genotypic characterization. 

The minimal inhibitory concentrations (MICs) of the tested antimicrobial substances determined for *K. pneumoniae* isolates are summarized in Table 1. Antimicrobial susceptibility testing (AST) results confirmed all isolates as ESBL producers. The recovered *K. pneumoniae* (n = 5) exhibited non-wildtype phenotypes (resistance) against ampicillin, cefotaxime, ceftazidime, cefepime and tetracycline (Table 1). In addition, AST showed that almost all strains displayed resistance against trimethoprim (4/5) and sulfamethoxazole (4/5). With regard to fluoroquinolone, a high rate of isolates was determined to be resistant to ciprofloxacin (n = 4) and nalidixic acid (n = 3), while low resistant rates were determined for gentamicin (n = 1), azithromycin (n = 2) and chloramphenicol (n = 1). However, all of the strains were susceptible to imipenem, meropenem, ertapenem, tigecycline, temocillin, colistin and cefoxitin (Table 1).

**Figure 1:**
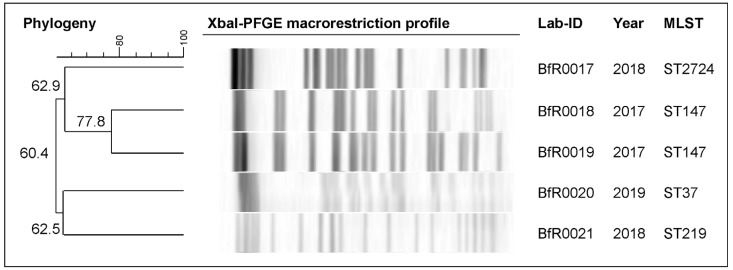
*XbaI*-PFGE dendrogram for *K. pneumoniae* isolates. The scale indicates the percentage of identity.

The differences in the AST results among the isolates were also confirmed by the prediction of phylogenetic relationships using *XbaI*-PFGE macrorestriction profiling. All isolates were assigned to distinct restriction profiles using a similarity cutoff value of 80% (Figure 1).

For in silico-based typing purposes, the genomic DNA of the isolates was subjected to WGS. The general features of the *K. pneumoniae* genomes are summarized in Supplementary Table S2. The overall G+C content of the genome was shown to range between 57.26 and 57.44%. The genomes were found to range between 5.345 and 5.465 Mbp in length and to contain from 5218 to 5388 coding sequences (genes). The WGS data revealed that the isolates belong to four different sequence types (ST). While the ST147 sequence type was assigned to two of the isolates, the remaining isolates were identified as members of the ST37, ST219 and ST2424 sequence type groups.

### 2.2. Acquired AMR and Single-Nucleotide Variations in the Chromosome 

In silico analysis of these isolates’ WGS data revealed the presence of known AMR sequences in the NCBI AMRFinder database. Of the isolates, all carried *bla*_CTX-M-15_, three carried *bla*_OXA-1_, and three carried *bla*_TEM-1b_. Additionally, all *K. pneumoniae* isolates harbored one yet-unassigned *bla*_SHV_ variant, varying in its coding sequence with respect to the sequences of the reference genes (Supplementary Table S3). 

The genes *sul2* and *sul1* were detected in five and one *K. pneumoniae* isolates, respectively, which were found to be resistant to sulphonamides. Based on the database, trimethoprim resistance was shown to be associated with the *dfrA*14 gene in three isolates and with the *dfrA*12 gene in one isolate. Tetracycline resistance (MICs ≥ 8) was observed in all *K. pneumoniae* isolates, and this resistance was associated with the presence of the *tet*(A) (n = 4) and *tet*(D) genes (n = 1, Supplementary Table S3).

All of the isolates were identified to carry the *oqxA* and *oqxB* genes, whereas the *aac*(6′)-lb-cr, *qnrB*1 and *qnrS*1 genes were identified in three, two and one isolates, respectively. WGS also confirmed that the isolates harbored aminoglycoside resistance genes, namely, *aac*(3)-lle, *aph*(3″)-Ib, *aph*(3′)-Ia, *aadA*2 and *aph*(6)-Id. Although fosfomycin MICs were not determined using the Sensititre panel, all isolates contained the *fosA* gene. Three isolates, for which the in vitro susceptibility results revealed no resistance to chloramphenicol, were positive for partial sequences of the *catB*3 gene.

**Figure 2:**
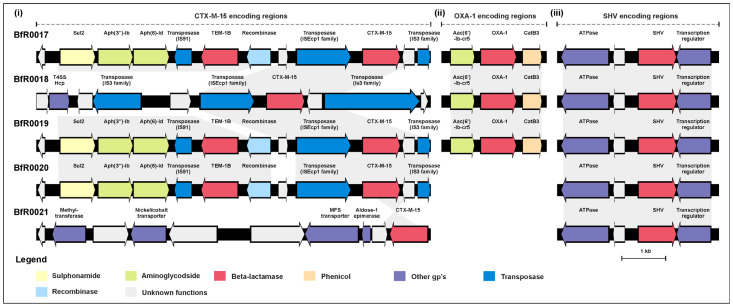
Organization of the independent genetic backgrounds comprising the (**i**) CTX-M-15, (**ii**) OXA-1 and (**iii**) SVH enzyme-encoding regions in the *K. pneumoniae* isolates. Arrows indicate the presence of the predicted coding sequences (CDS). Functional prediction of the gene products is indicated in the figure. DNA regions exhibiting sequence identity of >90% between the genomes are linked in grey.

Further dissection of the genetic background of the ESBL-encoding determinants showed the distribution of the *bla*_CTX-M-15_, *bla*_OXA-1_ and *bla*_SHV_ coding regions in distinct regions of the isolates’ genomes. Interestingly, the CTX-M-15-encoding region was found to be highly conserved, as the *bla*_CTX-M-15_ gene was detected in association with an ISEcp1 family transposon at the same site within the genome of the isolates. Only for the isolate BfR0021, the presence of this gene on a flanking site could be verified, as the gene was located on one end of the genomic contigs (Figure 2i). In addition to *bla*_CTX-M-15_, three isolates also carried further resistance genes (*bla*_TEM-1B_, *aph*(3″)-Ib, *aph*(6)-Id and *sul2*), which appeared to be flanked by further transposases/recombinases. For the remaining two isolates, no further resistance genes could be detected in close association with *bla*_CTX-M-15_. The second beta-lactam resistance-encoding region (Figure 2ii), containing the *bla*_OXA-1_ gene, was shown to be associated with *aac*(6′)-lb-cr5 and a partial *catB*3 gene. Interestingly, all three isolates carrying *bla*_OXA-1_ exhibited the same genetic organization of this resistance region. In the third region, all isolates carried a yet unassigned *bla*_SHV_ variant (Figure 2iii). 

The genes encoding outer-membrane proteins (OmpK35, OmpK36 and OmpK37), which were associated with cephalosporin resistance, were detected in all of the five isolates according to the ResFinder results. We also identified mutations in the *ompK*36 and *ompK*37 genes (Supplemental Table S4). In addition, seven alterations in the *acrR* gene appeared to be conserved in the genomes of the five isolates conferring resistance to fluoroquinolones. Furthermore, the ST147 isolates were found to carry also the GyrB (S83I) and ParC (S80I) alterations, affecting the fluoroquinolone tolerance of the BfR00018 and BfR00019 isolates.

WGS analysis also revealed the presence of diverse metal resistance systems including that providing resistance to copper (*pcoABCDRS*; n = 3), the copper/silver efflux transporter (*silABCEFPRS*; n = 3) and systems providing resistance to tellurium (*terBCDE*; n = 1) and arsenic (*arsADR*; n = 1) (Supplementary Table S5). Moreover, *K. pneumoniae* BfR0021 also contained the quaternary ammonium compound (QAC) efflux transporter gene *qacE*∆1. 

### 2.3. Virulence Factors of K. pneumoniae 

Using the WGS data, virulence factors in the isolates were also analyzed according to the virulence genes present in publicly available databases. The synthesis of enterobactin-related genes (*ent* and *fep*) was identified in all isolates. The virulence genes *yagW*/*ecpA*, *yagW*/*ecpB*, *yagW*/*ecpC*, *yagW*/*ecpD*, *ompA* and *ykgK*/*ecpR* for adhesion were detected in all *K. pneumoniae* isolates. Another virulence gene, *febG*, was detected in three isolates. In addition, one ST147 isolate (BfR0018) carried the highest number of genes (n = 22), including a gene cluster (the *ybt*–*irp* complex) encoding yersiniabactin, and also harbored the gene *fyu* related to yersiniabactin (Supplementary Table S6). 

### 2.4. Transmissibility of the bla_CTX-M-15_ Determinants 

By the use of in vitro filter mating for the evaluation of ESBL gene transfer, we were able to obtain beta-lactam-resistant transconjugants of the sodium azide-resistant *E. coli* strain J53, used as a recipient. Overall, high transfer frequencies ranging between 1.8 × 10^1^ (BfR0017) and 1.3 × 10^2^ (BfR0021) were obtained after mating at 37 °C for 24 h. Five individual transconjugants per isolate (BfR0017 to BfR0021) were further subjected to PCR for *bla*_CTX-M_ gene detection, confirming the presence of determinants encoding the CTX-M-15 enzyme. In addition, AMR testing on LB agar supplemented with selected antimicrobial substances (i.e., streptomycin and suphamethoxazole) resulted in the confirmation of the resistance results for BfR0017, BfR0019 and BfR0020; for the remaining transconjugants of the isolates, no further antimicrobial resistance was detected. 

### 2.5. Phylogenetic Comparison of ESBL-Producing K. pneumoniae from Chicken Meat and Human Infections

**Figure 3:**
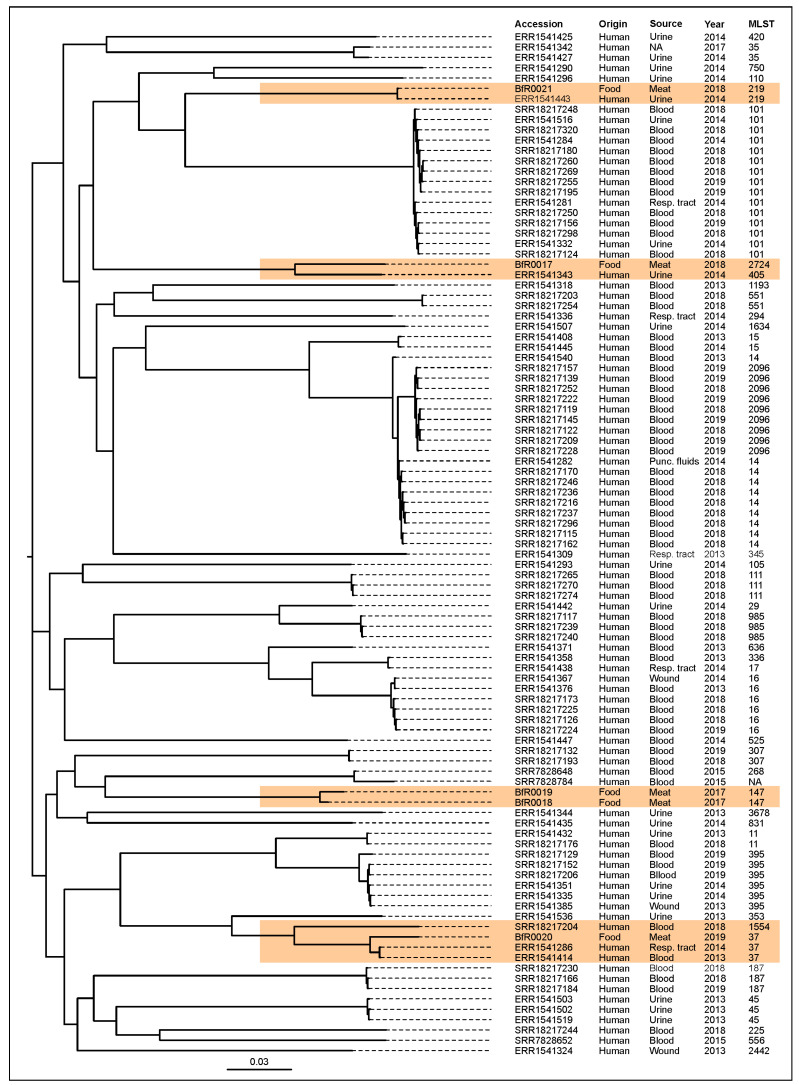
Phylogenetic relationship between genome sequences of *K. pneumoniae* chicken meat isolates and genome sequences of isolates from human infections in Türkiye. Genomes clustering with reference sequences of the database are indicated with orange.

To evaluate the impact of the recovered isolates, the WGS data of their genomes were used for a phylogenetic comparison. A subset of genome sequences of isolates from human infections (recovered from the bloodstream, urine, wounds or the respiratory tract), obtained from Turkish patients, were used for SNP tree generation. The results are shown in Figure 3. Surprisingly, all isolates showed a close relationship to previously described *K. pneumoniae* strains recovered from urine, blood and the respiratory tract. Especially, for the isolates BfR0017, BfR0020 and BfR0021, closely related genomes of human isolates were identified, suggesting that also underreported sequence types (ST2724, ST219 and ST37) may pose a threat to human health. 

## 3. Discussion

The prevalence of ESBL-producing *K. pneumoniae* has been reported to be high in humans, but seems to remain low in animals, to date. However, the emergence of ESBL-producing *K. pneumoniae* in animals raises concerns, in particular with regard to the risk of resistance transfer to human strains via the food chain. In this study, the overall occurrence of ESLB-producing *K. pneumoniae* in raw chicken meat samples was 2.5% in Türkiye, which is noticeably lower than the value we found for ESLB-producing *E. coli* (rate between 83% and 86%) in retail chicken meat samples, recently [21,22]. Hiroi et al. (2012) also reported that ESBL-producing *K. pneumoniae* colonized 3% of healthy chickens in Japan [23]. However, the occurrence rate of ESBL-producing *K. pneumoniae* in foods of animal origins varies greatly, ranging from 3.7% in chicken liver samples in Algeria [24] to 7.7% in retail chicken meat samples in the Netherlands [13,16], 23.4% in raw milk samples in Lebanon [10] and 25% in food fish samples in India [25]. 

In the current study, the majority of *K. pneumoniae* isolates displayed the MDR phenotype, expressing resistance to at least three different antimicrobial classes. Besides cephalosporin resistance, a high rate of resistance to tetracycline and ciprofloxacin was observed. High rates of resistance were also reported in various international studies. For example, previous studies in Spain and Germany also showed a high fluoroquinolone resistance in ESBL-producing *K. pneumoniae* from foods of animal origins and wastewater from chicken slaughterhouses [15,26,27,28]. In another study from the U.S.A., *K. pneumoniae* isolated from different meat sources in retail outlets frequently showed resistance to tetracycline and aminoglycoside antibiotics, and this situation was attributed to the extensive use of these antibiotics in livestock [29]. Contrary to these finding, ESBL-producing *K. pneumoniae* had a low level of resistance to aminoglycoside antibiotics in the current study, and this difference might have resulted from disparities in antimicrobial usage in animal production in different countries. 

In the present study, *XbaI*-PFGE verified that the examined ESBL-producing *K. pneumoniae* isolates were genetically diverse. The five isolates were assigned to four STs , with ST147 being the dominant one; the other sequence types were ST37, ST219 and ST2424. Recently, ST37 was also reported frequently in other studies of human clinical samples [30] and foods of animal origin (e.g., chicken meat) in Europe [31]. The ST147 isolate has recently emerged as one of the most frequently obtained clones from human clinical samples [32,33,34,35,36], and a recent systematic review thus recognized ST147 as a “high-risk clone” [37]. For example, a Hungarian study found that over 20% of ESBL-producing *K. pneumoniae* isolates belonged to the ST147 sequence type group [32]. A previous study also reported that a CTX-M-producing ST147 isolate was obtained from human clinical samples in Türkiye [38]. This clone was also detected in companion animals [39], livestock [26] and environmental samples [34]. Also the carbapenemase-producing *K. pneumoniae* ST219 type was identified in human and environmental isolates, as reported in the literature [40]. Additionally, we also identified a high level of sequence similarity between the ST2724, ST219 and ST37 genomes and the genomes of *K. pneumoniae* strains isolated from human clinical samples. 

Similar to previous reports using different *K. pneumoniae* sources [20,40], all *K. pneumoniae* isolates contained *bla*_CTX-M-15_ together with *bla*_SHV_ variants. Aminoglycoside (*aph*(3″)-Ib and *aph*(6)-Id) and tetracycline antimicrobial resistance genes (*tetA*) showed a relatively high prevalence in the isolates, despite the detection of low levels of gentamycin resistance. These five ESBL-producing *K. pneumoniae* isolates carried the *fosA* gene, encoding fosfomycin resistance. Recent studies carried out by Surleac et al. (2020) and Savin et al. (2022) also showed that 99.7–100% of *K. pneumoniae* isolates from clinical and wastewater sources exhibited *fosA*, which thus seems to be a common resistance determinant in *K. pneumoniae* [15,40].

Notably, previous studies revealed that the outer-membrane proteins OmpK35, OmpK36 and OmpK37 are associated with carbapenem resistance. Notably, deletion of *ompK*35 and mutation of *ompK*35, *ompK*36 and *ompK*37 were shown to lead to a significant increase in carbapenem resistance [41,42,43]. Özad Düzgün (2021) also identified several mutations in *ompK*36 and *ompK*37 in KPC-3-producing *K. pneumoniae* strains in Türkiye [38]. Our isolates possessed the genes *ompK*35, *ompK*36 and *ompK*37 and also had mutations in the latter two genes, without expressing phenotypic resistance to any of the tested carbapenem antibiotics, indicating that the mutations were recessive. In the current study, a variety of metal resistance gene clusters were identified in the isolates, while one isolate additionally possessed the QAC resistance gene *qacE*∆, which is a finding of great importance, as QAC-related genes are mostly found to be co-located on transmissible plasmids with antimicrobial resistance genes [44]. The QAC resistance gene might have been selected due to the application of disinfectants in the poultry production chain, since Surleac et al. (2020) recently found that strains of *K. pneumoniae* from wastewater carried the *qacE*∆ gene much more frequently than clinical isolates, attributing this finding to the selective pressure of disinfectants in wastewater [40].

Accumulating evidence revealed that siderophore production plays an important role in the pathogenicity of *K. pneumoniae* [45,46]. Aerobactin and yersiniabactin have been described as high virulence markers [45,46,47,48]. Although all isolates were found to harbor enterobactin-related genes, only one ST147 isolate carried the yersiniabactin gene. These findings are in accordance with previously published data [49,50,51], which noted that enterobactin gene markers are highly conserved in isolates. There is also information showing that yersiniabactin genes are rarely detected and hypervirulence-related serotypes are mostly absent in human isolates [50,51]. However, none of the genes associated with aerobactin or other hypervirulence-associated genes (i.e., *rmpA*/*rmpA*2) and K1/K2 capsule serotypes were detected in our isolates. 

*K. pneumoniae* is considered a highly important human pathogen and has been isolated with increased frequency from foods of animal origins. In this study, ESBL-producing *K. pneumoniae* strains isolated from raw chicken meat were assessed through whole-genome sequencing, and their genetic relatedness to strains originated from human clinical samples was determined. This study clearly highlighted that the high-risk clone ST147 is often present in contaminated chicken meat, indicating the importance of “One Health”-related studies to deal with this growing concern.

## 4. Materials and Methods

### 4.1. Sample Collection and K. pneumoniae Isolation 

Between June 2017 and June 2019, five non-repetitive *K. pneumoniae* isolates resistant to cephalosporins were obtained from raw chicken meat samples (n = 200), which were collected from supermarkets and butchers from different provinces in Türkiye. In the laboratory, the individual chicken meat samples (about 25 g) were homogenized in 225 mL of buffered peptone water and incubated at 37 °C overnight. After enrichment, loopfuls of the suspension were cultured on CHROMID^®^ ESBL agar (Biomerieux, Marcy l’Étoile, France) and incubated at 37 °C for about 24 h. On ESBL agar, non-metallic-green-sheen colonies considered as non-*Escherichia* were subcultured on blood agar medium overnight to obtain individual pure colonies. Species identification was carried out using mass spectrometry (MALDI-ToF MS Biotyper; Bruker GmbH, Bremen, Germany) with the direct transfer method, according to the recommendations of the manufacturer. 

### 4.2. Antimicrobial Susceptibility Testing (AST) Using Broth Microdilution 

The in vitro antimicrobial susceptibility of the isolates was determined using broth microdilution with the Sensititre™ test panels (EUVSEC/EUVSEC2; Thermo Fisher Scientific, Waltham, MA, USA). All isolates were tested for ampicillin, azithromycin, cefepime, ciprofloxacin, colistin, ertapenem, cefoxitin, gentamicin, imipenem, meropenem, nalidixic acid, cefotaxime, ceftazidime, temocillin, tetracycline, tigecycline, trimethoprim, chloramphenicol, sulfamethoxazole, cefotaxime/clavulanic acid and ceftazidime/clavulanic acid susceptibility. AST was conducted according to the recommendation of EUCAST, and the breakpoints suggested for the clinical cut-off values [52] were used for the interpretation of the results. 

### 4.3. Pulsed-Field Gel Electrophoresis (PFGE) for Macrorestriction Profiling 

PFGE was performed using the *XbaI* restriction endonuclease (Thermo Scientific, Schwerte, Germany) for macrorestriction profiling and clonal relationship determination. Agarose plugs were prepared and separated by electrophoresis on a 1.5% (*w*/*v*) agarose gel according to the established PulseNet protocol (https://www.cdc.gov/pulsenet/pdf/ecoli-shigella-salmonella-pfge-protocol-508c.pdf, accessed on 31 July 2021). The *Salmonella* Braenderup strain H9812 was used as a reference marker, and the restriction profiles were analyzed using BioNumerics, (version 7.0; Applied Maths, Sint-Martens-Latem, Belgium).

### 4.4. In Vitro Conjugation Assay

To determine the potential transmissibility of the ESBL resistance, the individual isolates were subjected to in vitro filter mating studies by using the sodium azide-resistant *E. coli* strain J53 as a recipient. Filter mating studies were conducted for 24 h at 37 °C on solid lysogeny broth (LB) agar, as previously described [53]. Selective cultivation of transconjugants was conducted on LB agar supplemented with cefotaxime (1 mg/L) and sodium azide (100 mg/L). The transfer frequency of the ESBL resistance-conferring plasmid was calculated by determining the ratio of transconjugants per donor cell. The size of the CTX-M-carrying plasmids in the parental strains and the transconjugants was determined by PCR and AST, as previously described [54].

### 4.5. WGS and Bioinformatics Analyses

Genomic DNA extraction for DNA sequencing library preparation was conducted using the PureLink Genomic DNA Extraction Kit (Invitrogen/Thermo Scientific, Schwerte, Germany) according to the manufacturer’s instructions. Short-read, paired-end whole-genome sequencing (WGS) was performed on all isolates using an Illumina benchtop device, as specified by the manufacturer (Illumina, San Diego, CA, USA) [55]. The quality of the raw sequencing data was evaluated using the Aquamis pipeline [56]. The sequencing data were subjected to the Bakcharak pipeline (https://gitlab.com/bfr_bioinformatics/bakcharak, accessed on 31 July 2023), which uses NCBI AMRFinder [57] for the detection of antimicrobial resistance determinants. It also includes ABRicate (https://github.com/tseemann/abricate, accessed on 31 July 2023) for the detection of plasmid replicon sequences from the plasmidfinder database [58], as well as virulence genes from the VFDB [59]. MLST typing was performed using mlst (https://github.com/tseemann/mlst, accessed on 30 November 2023) and the PUBMLST schemes [60]. Basic bioinformatics analysis was performed using DS Gene (version 2.5, Accelrys Inc., San Diego, CA, USA). For a detailed analysis, further in silico typing was conducted using selected tools (i.e., ResFinder (v4.1; default settings), CSIphylogeny of the Center for Genomic Epidemiology (https://www.genomicepidemiology.org/, accessed on 30 November 2023), as specified. CSIphylogeny (v1.4; default settings) was used for the generation of the SNP-based phylogenic tree. The resulting Newick file was subjected to FigTree (v1.4.4, default settings)) for illustration. 

The nucleotide sequence data of the *K. pneumoniae* isolate de novo assemblies are deposited in Genbank under the accession numbers: JAIPRD000000000 (2019_OU_BfR0017), JAIPRF000000000 (2019_OU_BfR0018), JAIPRE000000000 (2019_OU_BfR0019), JAIPRG000000000 (2019_OU_BfR0020) and JAIPRH000000000 (2019_OU_BfR0021).

## Figures and Tables

**Table 1:** MIC determination for *K. pneumoniae* isolates by AST.

Lab ID	AMP	TRM	CIP	NAL	GEN	AZI	TET	TGC	COL	CHL	TMP	SMX
BfR0017	>64	8	2	16	32	32	>64	0.5	≤1	≤8	>32	>1024
BfR0018	>64	4	>8	>128	1	16	>64	0.5	≤1	≤8	≤0.25	≤8
BfR0019	>64	4	>8	>128	1	16	>64	1	≤1	16	>32	>1024
BfR0020	>64	4	0.06	≤4	≤0.5	16	>64	0.5	≤1	≤8	>32	>1024
BfR0021	>64	4	1	≤4	≤0.5	64	>64	0.5	≤1	≤8	>32	>1024
**Lab ID**	**FEB**	**FOT**	**TAZ**		**FOX**	**ETP**		**IMI**	**MER**	**TAX/CLA**	**TAZ/CLA**	
BfR0017	8	>64	8		4	0.03		0.25	0.06	≤0.06	0.25	
BfR0018	16	>64	16		8	0.12		0.5	0.12	≤0.06	≤0.12	
BfR0019	16	>64	8		4	0.03		1	0.06	≤0.06	≤0.12	
BfR0020	8	64	8		4	0.03		0.25	≤0.03	≤0.06	≤0.25	
BfR0021	32	>64	16		2	0.03		0.25	≤0.03	≤0.06	0.5	

In grey, isolates exhibiting MIC leading to phenotypic resistance are indicated. MIC values are given in mg/L. Abbreviations: ampicillin (AMP), azithromycin (AZI), cefepime (FEP), chloramphenicol (CHL), ciprofloxacin (CIP), colistin (COL), ertapenem (ETP), cefotaxime (FOT), cefoxitin (FOX), gentamicin (GEN), imipenem (IMP), meropenem (MERO), nalidixic acid (NAL), sulfamethoxazole (SMX), cefotaxime/clavulanic acid (TAX/CLA), ceftazidime (TAZ), ceftazidime/clavulanic acid (TAZ/CLA), tetracycline (TET), tigecycline (TGC), trimethoprim (TMP), temocillin (TRM) and sulfamethoxazole (SMX).

## Data Availability

Experimental data are provided in the manuscript and the supplemental material. The WGS data of the analyzed *K. pneumoniae* genomes were deposited in Genbank and are available via the accession numbers mentioned in the manuscript.

## Supplementary Materials

The following supporting information can be downloaded at https://www.mdpi.com/article/10.3390/antibiotics13010014/s1. Table S1: General information on the *Klebsiella pneumoniae* isolates examined in this study; Table S2: Whole-genome sequencing results of ESBL-producing *Klebsiella pneumoniae* isolates. Table S3: Acquired antimicrobial resistance identified in silico; Table S4: Point mutations associated with antimicrobial resistance identified in silico; Table S5: Metal and disinfectant resistance genes; Table S6: Virulence genes.
